# The Use of Unmanned Aerial Vehicles in Remote Sensing Systems †

**DOI:** 10.3390/s20072003

**Published:** 2020-04-03

**Authors:** Aleksander Olejnik, Łukasz Kiszkowiak, Robert Rogólski, Grzegorz Chmaj, Michał Radomski, Maciej Majcher, Łukasz Omen

**Affiliations:** 1 Faculty of Mechatronics & Aerospace, Military University of Technology, 00-908 Warsaw, Poland; aleksander.olejnik@wat.edu.pl (A.O.); robert.rogolski@wat.edu.pl (R.R.); michal.radomski@wat.edu.pl (M.R.); maciej.majcher@wat.edu.pl (M.M.); lukasz.omen@wat.edu.pl (Ł.O.); 2 DRI Solutions Sp. z o.o, 00-580 Warsaw, Poland; office@drisolutions.eu

**Keywords:** remote sensing, UAV, helicopter, image acquisition, precise flight

## Abstract

This paper describes the possibility of using a small autonomous helicopter to perform tasks using a remote sensing system. This article further shows the most effective way to properly set up autopilot and to process its validation during flight tests. The most important components of the remote sensing system are described and the possibilities of using this system to monitor gas transmission and distribution networks are presented.

## 1. Introduction

Remote sensing refers to the process of detecting and monitoring the physical characteristics of an area by measuring reflected and emitted radiation at a certain distance from the targeted area. In general, remote sensing applies to all measurements that are conducted remotely. Remote sensing methods are divided into active and passive, whereby active remote sensing describes when a signal is sent from the sensor and is reflected back from the object to be received and analyzed, whereas passive remote sensing methods are based on the analysis of signals emitted by the observed object [1,2]. Satellites and manned aircrafts that are used to collect data remotely are effective but relatively expensive, and these technologies tend to have low spatial and temporal resolutions. They also tend not to be flexible enough to accommodate the needs of many organizations working with these applications [3,4]. To overcome these limitations, unmanned aerial vehicle (UAV) technologies have been developed for remote sensing applications. The main advantage of UAVs is that they can be used in high-risk situations without endangering human life. UAVs can also be utilized in inaccessible areas, at low altitude, and at flight profiles close to the objects where manned systems cannot be flown [5]. In particular, unmanned helicopters have been investigated for several decades [6,7], with the rapid development of this type of flying object now being observed [8,9]. This paper describes the integration process of adapting an autonomous helicopter to perform data gathering in a remote manner. Such tasks require precise flights, for example, through predefined waypoints, while maintaining a given flight speed and altitude. So-called stable flights are especially important when a platform is flying very close to the ground, i.e., 2−3 meters high. In many cases, some additional above-ground level (AGL) sensors are required to provide accurate distance to ground in autopilot [10,11].

The following sections of this paper describe the main components of a helicopter-based remote sensing vehicle that was developed by the Military University of Technology in Poland. Moreover, this paper presents the most effective way of autopilot configuration and the process of its validation during flight tests. Section 2 contains a description of the proposed system in terms of its fields of application. Section 3 presents the obtained results, and the last sections conclude the paper with a discussion. In comparison to the paper entitled *Precise Remote Sensing Using Unmanned Helicopter* that was presented during the 2019 IEEE 5th International Workshop on Metrology for AeroSpace (MetroAeroSpace) held in Torino in June 2019 [1], this paper contains the latest research data from flight tests and a more detailed description of the proposed uses of the developed system. One of the most valuable parts of this paper includes a detailed description of the work performed to achieve in-flight UAV stability at the level allowing for the detection, recognition, and identification of a person from 500 m.

## 2. Materials and Methods

### 2.1. General Description of UAV WABIK

**Figure 1:**
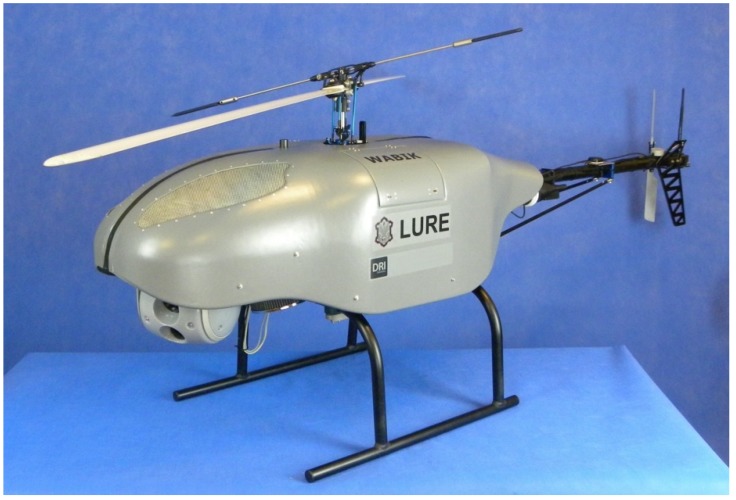
Unmanned aerial vehicle (UAV) WABIK.

The main element of developed remote sensing system is the unmanned aerial vehicle WABIK (English: LURE), shown in Figure 1, a 35 kg autonomous helicopter with a 12 kg payload capacity. WABIK is driven by a 9 HP two-stroke piston engine. The basic flight time with standard fuel tanks is 60 minutes, which can be doubled after installing additional fuel tanks. The maximum flight speed in autonomous mode is 35 m/s, the maximum vertical take-off speed is 8 m/s and the maximum flight altitude is 2000 m AMSL. WABIK was equipped with the MP2128Heli [12] autopilot enabling autonomous take-off, landing, and flight over predefined waypoints. Autopilot also allowed control of the system in semi-autonomous mode, where the movements of the RC radio stick were treated by the autopilot as simple commands, such as flying forward at a given speed, increasing the flight altitude, or landing, while autopilot dealt with flight stabilization.

According to the classification [13] shown in Table 1, alongside the maximum take-off weight (MTOW), the range, the duration of the flight, and the maximum ceiling, WABIK is classified in the mini-UAV category. WABIK was designed so that 5 kg of payload must be mounted at the front of the frame to achieve mechanical balance. Remote sensing from onboard payload is one of WABIK’s tasks. This payload could be an optoelectronic system used for observation and surveillance, a LIDAR system used to construct digital 3D representations of ground objects, a methane detection system used to detect leaking pipes or a spray system used for the agriculture industry [14,15].

### 2.2. Flight Control System

**Figure 2:**
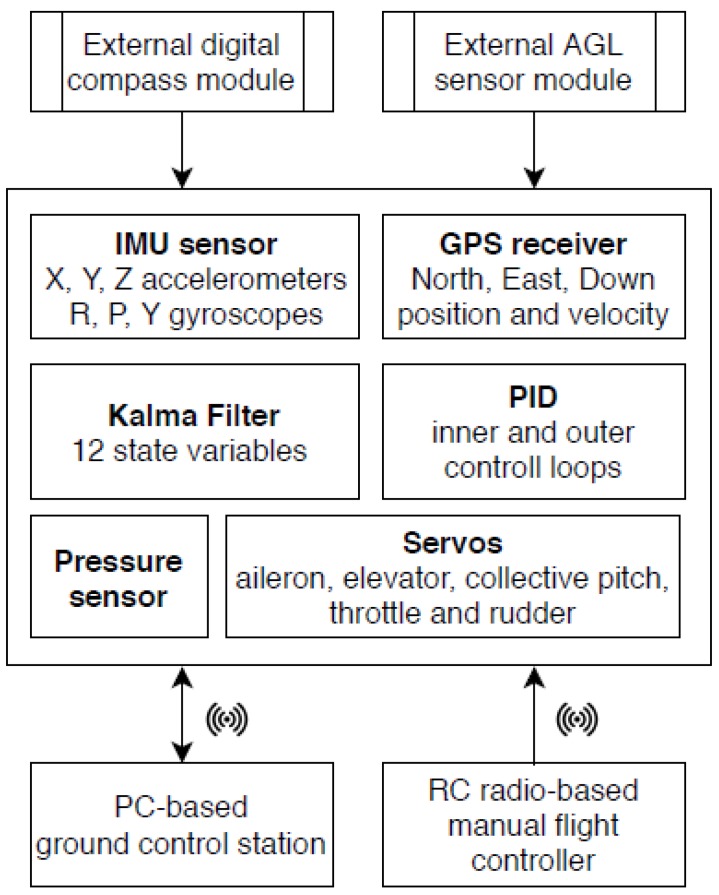
Flight control system architecture.

**Figure 3:**
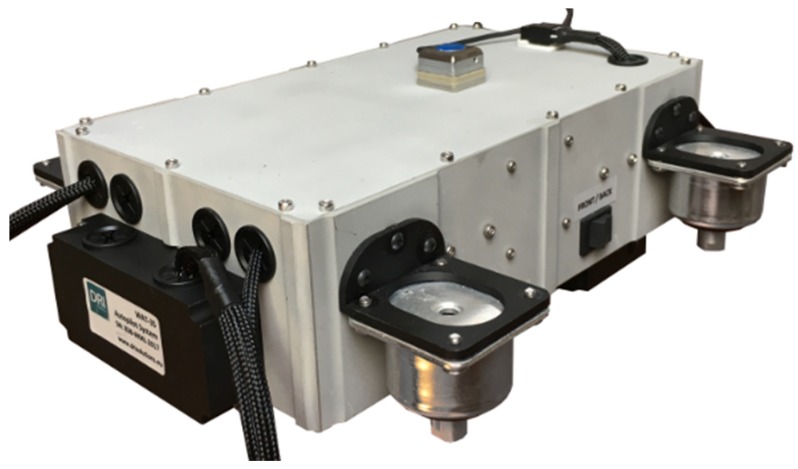
Autopilot installation.

The WABIK was equipped with the MP2128Heli [12] flight control system. The sensory equipment of the autopilot included a GPS receiver, an ultrasonic height sensor, a barometric sensor, a digital magnetic compass, and diagnostic sensors to monitor the temperature of the piston engine and the fuel levels in the tanks. The autopilot architecture and autopilot installation are shown in Figure 2 and Figure 3, respectively.

The MP2128Heli autopilot is a combination of a number of sensors, namely, the Global Navigation Satellite System (GNSS) software and a control system controlling aircraft actuators. The main task of this system is to estimate the following characteristics of the aerial vehicle [16]:

The position: Latitude, longitude, meters above sea level;

The speed: North speed, east speed, vertical speed;

The orientation: Roll, pitch, yaw.

In order to estimate these parameters, the autopilot was integrated with the below-mentioned sensors:

The Inertial Measurement Unit (IMU), a combination of gyro sensors and accelerometers measuring angular velocity and linear acceleration along the X, Y, Z axes and in the X, Y, Z directions, respectively;

The Attitude and Heading Reference System (AHRS), a combination of the IMU system with a magnetic earth field sensor providing information regarding the direction of flight;

The Global Navigation Satellite System (GNSS), which provides location, speed, and altitude information.

**Figure 4:**
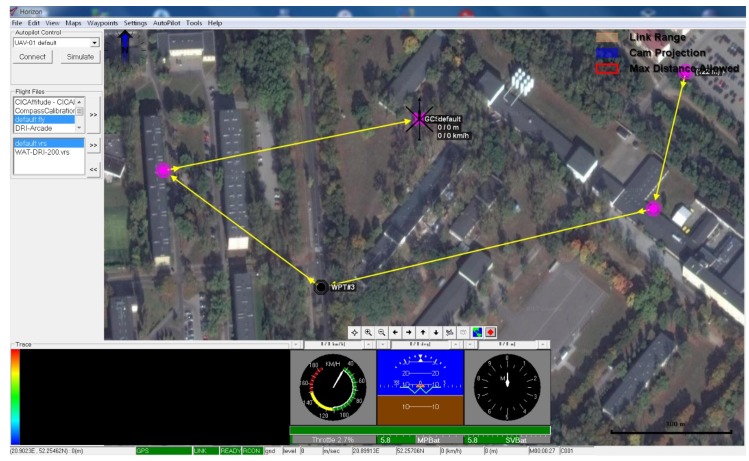
Horizon software interface used in flight control.

The use of GNSS in the autopilot system was carried out in a loosely connected configuration (loosely coupled INS/GPS) [17]. The WABIK flight control was made available to the operator through the Horizon software, as shown in Figure 4.

The autopilot contained six implemented PID controllers that were responsible for controlling various flight functions depending on the selected flight mode, including:

Controller 1—aileron from roll: Controlled aileron deflection to ensure that the difference between the specified pitch and the pitch estimated by the KF was minimal;

Controller 2—elevator from pitch: Controlled the rudder deflection to ensure that the difference between the specified slope and the slope estimated by the KF was minimal;

Controller 3—hover rudder from heading: Controlled the overall pitch of the tail rotor disc to ensure that the difference between the given direction of flight and the direction estimated by the KF was minimal;

Controller 4—throttle from altitude: Controlled the throttle deflection and the overall stroke of the control disc to ensure that the difference between the set flight altitude and the ceiling estimated by the KF was minimal;

Controller 5—hover pitch from X velocity: Controlled the rudder deflection to ensure that the difference between the set speed in the X direction and the speed estimated by the KF was minimal;

Controller 6—hover roll from Y velocity: Controlled the aileron deflection to ensure that the difference between the set speed in the Y direction and the speed estimated by the KF was minimal.

For example, when a helicopter hovered in a configured control type to maintain a set position, the autopilot set the desired speed in the X and Y directions to reach the set hover point (fixed GPS position set). Based on the difference between the set speed and the actual speed (estimated by the KF) in the X and Y directions, the required pitch and roll angles were used according to the input of the above-mentioned Controllers 1 and 2, respectively. All available control modes implemented in the autopilot and their connections with controllers used in given control modes are presented in Table 2 [16].

For example, in the CIC attitude control mode, changing the direction stick of the RC radio (corresponding to the change in the angle of attack of the tail blades) gave a new desired direction, and the flight control system was responsible for obtaining and maintaining the desired direction. The above control modes could also be combined with each other. For example, in the CIC altitude position control type, the right stick of the RC transmitter shifted the waypoint (directions X and Y) to a new desired position, with the flight control system ensuring that the WABIK system moved to that point. The left stick of the RC transmitter set a new flight altitude and new direction, while the flight control system was responsible for maintaining a new altitude, direction, and position. The CIC altitude velocity type was similar to the CIC altitude position type, except that the right stick on the RC radio changed the desired flight speed in the same way as the CIC velocity type.

The most advanced and most frequently used flight mode in WABIK is a fully autonomous flight with navigation points. The predefined flight route consisting of a number of waypoints, can be modified during the flight via the ground flight control station. The start of the autonomous flight is carried out by a button integrated into the ground base software in the HORIZON program [16].

### 2.3. Optoelectronic Equipment

**Figure 5:**
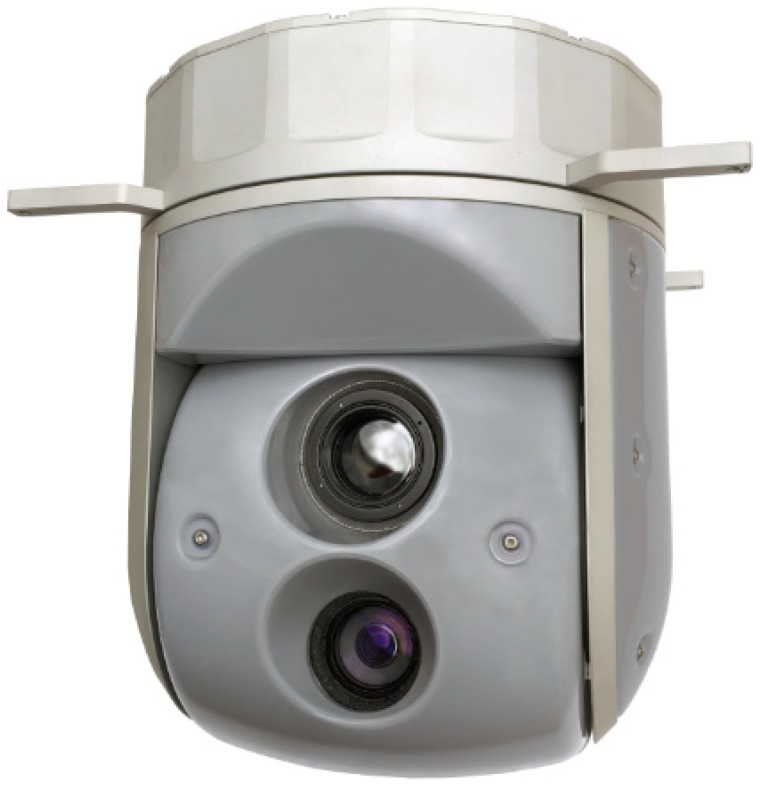
Optoelectronic head developed especially for use with WABIK.

WABIK is capable of carrying an 8” optoelectronic head equipped with a thermal imaging camera and a daylight camera, as shown in Figure 5. It allows remote detection, recognition, and identification and is equipped with a CCD day camera and a longwave infrared thermal camera with radiometry. Target tracking, geo-pointing, an 18x optical continuous zoom, radiometry and isotherm functionality, robust stabilization, and an integrated INS/GPS allow this system to be used in a wide range of military and industrial applications [18,19].

**Figure 6:**
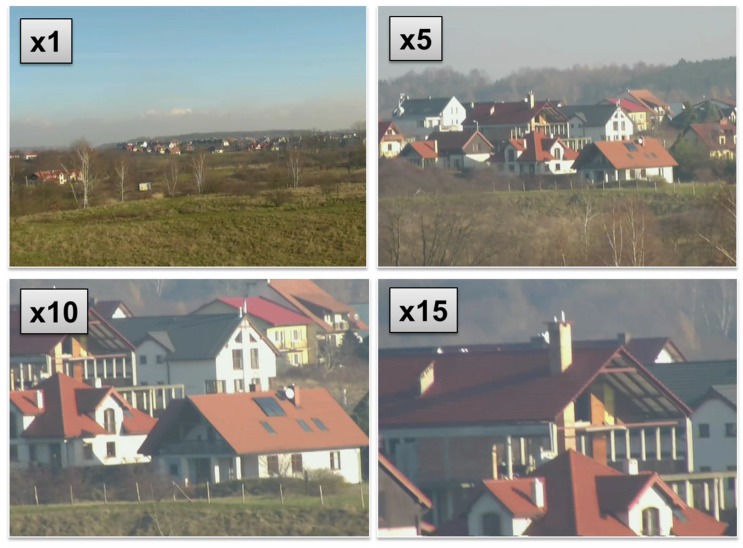
Real-time video stream from a daylight camera with a 15x optical zoom.

**Figure 7:**
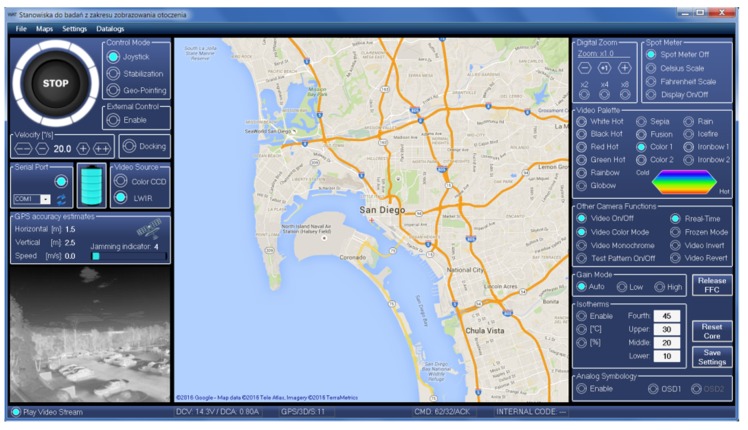
Optoelectronic head control software interface.

Examples of WABIK’s remote detection capability (500 m away from the target) using a daylight camera with a 15x optical zoom are presented in Figure 6. Full functionality of the developed optoelectronic head was made available to the operator through software installed at the ground flight control station, the interface of which is shown in Figure 7.

This software allows the following features:

Display of video transmitted in real time from an unmanned helicopter;

Control of the optical zoom;

Application of defined color palettes to the image;

Application of subtitles to the image (OSD);

Definition of isotherms or spot temperature measurements of the observed objects.

The video stream from the vision sensors (VIS and MWIR) was coded, compressed, and encrypted using a cryptographic module mounted on an unmanned helicopter. The video stream prepared in this way was sent via the TCP/IP protocol to the transmission module mounted on the helicopter, where it was streamed using Real Time Protocol (RTP). The ground base station was equipped with a receiving module, in which the process of decrypting and decoding the video signal was carried out.

The WABIK system was further equipped with three communication links. The first communication link operated in the UHF band and implemented a helicopter connection with the flight station ground base. The second communication link operated in the S band and was used for operating the on-board observation head. The third communication link was used for data transmission sent from a conventional RC radio to the autopilot.

## 3. Results

For UAVs based on helicopter airframes, it is critical to achieve stable hovering according to a given altitude, a stable tail during flight, and stable forward flight before installing any sensors onboard the vehicle. For instance, automated power line inspection tasks require the use of close-range sensors, e.g., thermal cameras, to detect broken insulators. Once an insulator is chosen for further inspection, the UAV hovers while maintaining the given position and altitude. Otherwise, the onboard payload must be additionally stabilized, for instance, by using gyroscopes, an active vibration isolation system, or drives. Maintaining a given speed of a UAV helicopter is much more complicated in comparison with the fix-wing method because helicopter autopilots are unable simply reduce the throttle to decrease speed. Further, autopilots must estimate and control three times more of a dynamic state compared to fix-wing. These states are controlled by feedback loops which are not in use simultaneously. Autopilot enables feedback loops as required to control different aspects of flight.

**Figure 8:**
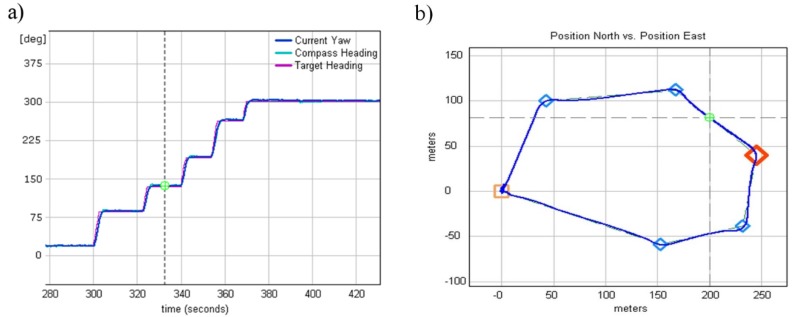
(**a**) Current and desired heading. (**b**) Predefined waypoints and achieved flight path.

Every single loop was adjusted during real terrain flights. The first rudder loop was adjusted as it controlled the most important part of helicopter, the tail boom. Due to the sensitivity of the tail rotor to wind, an additional external gyroscope was used to improve the tail rotor servo control task. This external yaw gyroscope was responsible for tail boom stabilization within ±0.5° (internal heading loop), while a second gyroscope acted as feedback sensor in an external heading loop responsible for controlling the changes of the helicopter course (heading). The rudder feedback loop was the most important and most challenging loop in terms of tuning because, if the rudder lost stability and started to oscillate or even increase the amplitude of the tail oscillation, it would have been difficult to recover the helicopter from such a state and may have caused a crash. An example heading loop performance during the fully autonomous flight through the predefined waypoints with a wind speed up to 10 m/s is presented in Figure 8.

**Figure 9:**
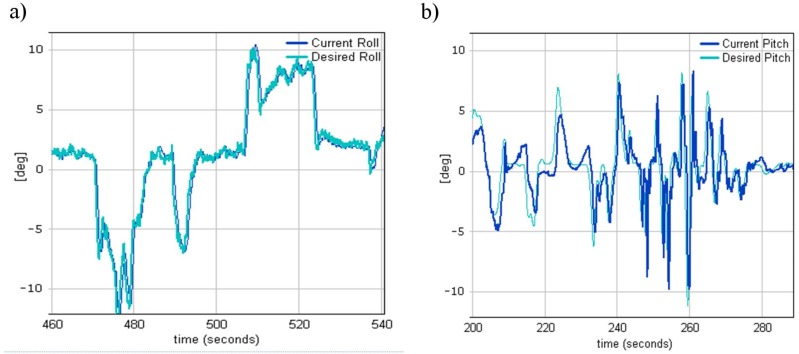
(**a**) Current and desired roll angle. (**b**) Current and desired pitch angle.

Once tail boom stability was achieved and no rudder oscillations were seen in data logs, the pitch and roll control loops were tuned. Pitch or elevator feedback loop controlled the longitudinal cyclic pitch to minimize the difference between the actual and the desired pitch, while the roll or aileron feedback loop controlled the lateral cyclic pitch to minimize the difference between the actual and the desired roll. These two loops were also called the inner loops as they controlled helicopter attitude in any aspect of flight (take off, hover, flight, or landing), while the outer loops utilized them to achieve the given forward speed, for instance. To start the tuning of the inner loops, the inner loops were isolated from the outer loops to simplify the adjusting procedure. This was done by enabling the arcade modes in the MP2128Heli autopilot. The arcade modes [12] are a set of hybrid control modes which allow a pilot to control a helicopter’s higher-level behavior without having to actually fly the helicopter. The MP2128Heli stabilizes the helicopter and manages the helicopter’s flight controls based on the higher-level inputs provided by the pilot. There are three main CIC arcade modes. To find the best P, I, and D terms for the abovementioned pitch and roll loops, maximum desired (±20°) input signals were applied from the RC transmitter during flight during CIC attitude arcade mode. The helicopter reactions (current roll and pitch) for the given inputs, along with the P, I, and D, terms were presented to the ground control station and analyzed in real time during the flight. This way, base values of the PID terms without causing attitude oscillations were quickly found only in few flights. Examples of the obtained results are illustrated in Figure 9.

As can be seen, better performance was achieved for the loop which controlled the helicopter roll angle (rotation about the X axis). This was due to the nature of the helicopter mass distribution, which was much more concentrated around the center of gravity on the Y axis than the X axis, which in turn required the application of more forces to make a helicopter pitch rotation.

**Figure 10:**
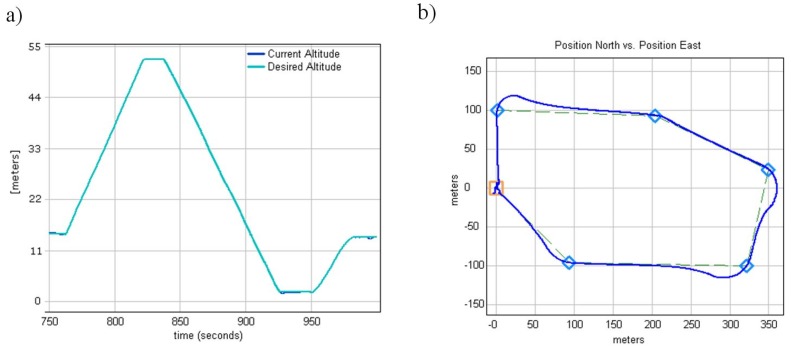
(**a**) Altitude loop performance. (**b**) Flight trajectory through waypoints.

At this stage of tests, only one internal loop was left to tune, i.e., the altitude loop responsible for controlling the throttle in response to the Z velocity. This loop was adjusted in the same manner as the previous loops. The desired altitude was set either by the RC transmitter stick or the ground control station. The response of the helicopter altitude was observed and analyzed at the ground control station and the PID terms were adjusted in the real time. The final results of the achieved altitude loop performance are shown in Figure 10a. The error between the current and desired altitudes did not exceed 0.2 m RMS.

During the next test flights, two external control loops responsible for maintaining helicopter velocity were tuned. These loops were “external” because they utilized two already tuned roll and pitch control loops. The first outer loop was the hover pitch from the X velocity and controlled the longitudinal cyclic to minimize the difference between the actual and the desired hover X velocity. The second loop was the hover roll from the Y velocity and controlled the lateral cyclic to minimize the difference between the actual and the desired hover Y velocity. For testing purposes, a flight trajectory, as shown in Figure 10b, was defined. The trajectory consisted of a few tight turns and a few smooth turns, which are more complicated maneuvers from a helicopter dynamic point of view. The constant flight speed was defined as 10 m/s.

**Figure 11:**
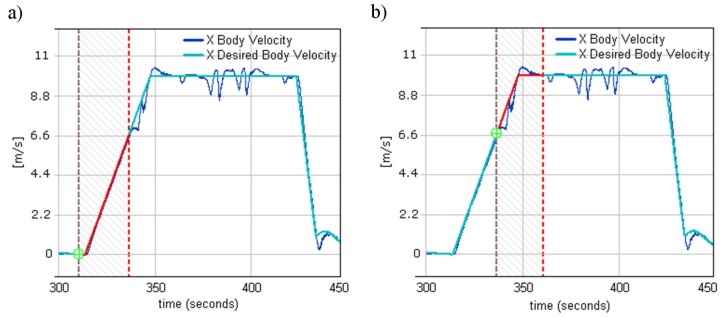
(**a**) X body velocity while approaching the 1st waypoint. (**b**) X body velocity while approaching the 2nd waypoint.

As seen in Figure 11a, the first part of the chart corresponding to flying from takeoff waypoints (point 0,0 in Figure 10b) to the first waypoint (point 0,100 in Figure 10b) was marked. During this part of the trajectory, two loops (X and Y body velocity control loops) were responsible for accelerating the helicopter from 0 to the given 10 m/s. Once 6.6 m/s was reached, small speed disturbances were visible as the helicopter entered the first turn. Similar speed disturbances were seen while the helicopter approached the second waypoint (Figure 11b). At this part of trajectory, the desired flight speed was accomplished. The worst speed disturbances were observed when the helicopter approached a tight turn (point 350, −25 in Figure 10b) flying at 10 m/s, followed quickly by the next tight turn. This case is illustrated in Figure 11a.

When fast helicopter deceleration was required, e.g., as determined by an anti-collision system, it was not possible to find PID terms that satisfied both the smooth velocity changes in forward flight and fast deceleration. This was due to a rotor head mechanical construction and the way it acted when the helicopter was flying forward and backward. Many tests were performed to find a compromise between these two requirements; however, when the helicopter was able to decelerate from, for instance, 10 m/s in 5 seconds, it was not able to perform smooth forward flight without pitching up and down (rotor head swashplate oscillation).

**Figure 12:**
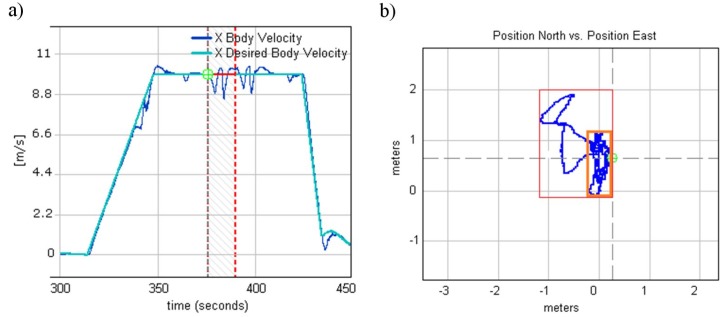
(**a**) X body velocity while approaching the 4th waypoint. (**b**) Relative position during autonomous takeoff.

After tuning all of the internal and external loops, the fully autonomous test flight was accomplished, which aimed to ascend according to a given climb speed of 6 m/s to a desired altitude of 20 m and then hover until a new command was received. As the position did not change, this maneuver theoretically should have been done in the same relative X and Y positions. Figure 12b displays the north vs. east relative positions. The relative positions during the test did not go beyond a 2 m × 1 m rectangle (marked in red). However, the GPS receiver CEP error, which was 2.5 m, should be kept in mind. The GPS position drifted when the helicopter was still on the ground, which is marked in orange in Figure 12. After the inner and outer feedback loops were tuned, WABIK was ready to receive installation of the optoelectronic head and perform one of its remote sensing tasks, namely, human detection and recognition.

## 4. Fields of Use

The developed monitoring system using vertical takeoff and landing unmanned aerial vehicles could be used to [15,19,20]:

Monitor gas transmission and distribution networks;

Patrol forests and act to recognize fires;

Monitor forest and agricultural degradation;

Function in air reconnaissance during floods and flooding;

Patrol border areas;

Surveil communication routes surveillance (railway traction and roads), particularly key transportation hubs;

Surveil special strategic importance land infrastructure (industrial complexes, power plants, airports, and seaports);

Monitor stadiums or outdoor events.

**Figure 13:**
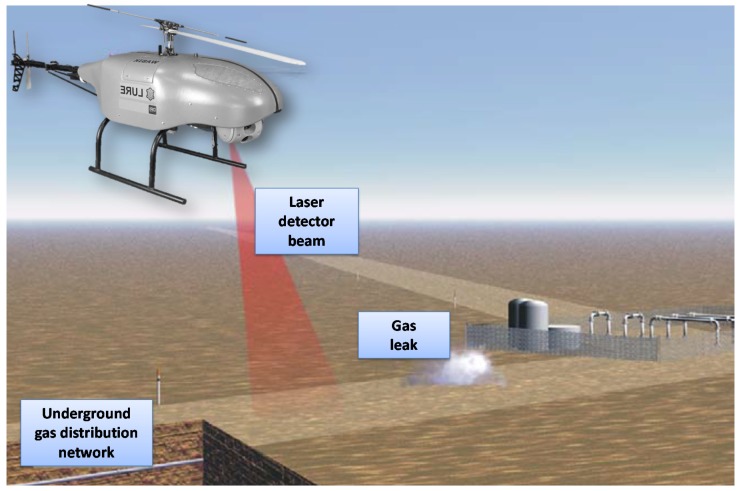
Remote sensing system in use to detect gas leaks from pipelines.

In the abovementioned situations, the use of unmanned aerial vehicles as discrete and non-invasive means of obtaining information seems to be expedient and the most reasonable solution. In regard to gas transmission network monitoring, the solution currently in development is aimed at assessing the technical condition of the infrastructure, monitoring the area in the immediate vicinity of the gas pipeline, and detecting possible damage. WABIK could be equipped with methane detectors (as shown in Figure 13) and, thanks to its small size and autonomous control system, it would be able to approach a gas pipeline at a distance that allows effective and reliable measurement.

**Figure 14:**
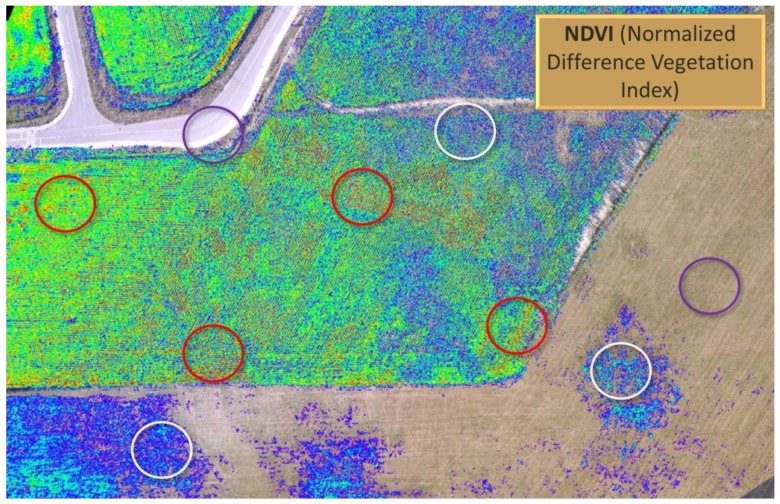
Remote sensing system in use to obtain high-quality multispectral imaging.

Thanks to its relatively big payload, WABIK is used in continuously developing remote sensing systems to obtain high-quality multispectral imaging. This special type of imaging is increasingly used in modern agriculture to obtain information regarding plant health and growth. Farm management decisions include information collected from remote sensing systems to increase crop productivity. Figure 14 shows an image of a Normalized Difference Vegetation Index (NDVI) distribution obtained using WABIK equipped with multispectral camera system. NDVI is a vegetation index used to quantify green vegetation. It normalizes green leaf scattering in the near infra-red wavelength and chlorophyll absorption in the red wavelength. NDVI is a measurement of the reflectivity of plants expressed as: the ratio of near-infrared reflectivity (NIR) minus red reflectivity (VIS) over NIR plus VIS:

(1) $$NDVI=\frac{NIR-VIS}{NIR+VIS}$$

Figure 14 shows red circles, indicating corn field areas in worse condition, and white circles, indicating crops in very poor condition. Objects that are not plants are indicated blue circles. Therefore, NDVI is a simple way to measure general plant health.

Measurements using the multispectral camera were carried out using recordings of the accurate aircraft position.

## 5. Discussion

The progress of miniaturization of electronics and the continuous development of robotics has allowed for the development of unmanned aerial vehicles, which can successfully perform tasks that, until now, were reserved for manned platforms. The use of UAV’s in remote sensing systems allows us to significantly eliminate human errors. Until now, many people were involved in data acquisition, e.g., as pilots, observers, aircraft maintenance unit members, or data processing specialists. The use of unmanned aerial vehicles will automate this process, making it much cheaper and more reliable. Economically this is also justified, because the operation of manned helicopters is several times more expensive compared to the operation and maintenance of small unmanned helicopters.

The effective operation of the monitoring system directly depends on the functionality of its individual components. Therefore, during the development process of vertical takeoff and landing platform, particular emphasis was placed on ensuring its high functionality. Achieved in-flight WABIK stability approved by data analysis, as presented in Figure 9, Figure 10 and Figure 11, allowed target observation by an onboard optoelectronic head, resulting in detection, recognition, and identification. WABIK can also integrate quickly and easily with many others sensors, like thermal imaging cameras, hyperspectral cameras, or a specialized system designed to monitor natural gas transmission and distribution networks. High-quality multispectral imaging can support agriculture (Figure 14) and can also be used during natural disasters for localization of water bodies. Current solutions to monitor gas transmission and distribution networks require the use of manned helicopters with specially trained pilots, who identify gas leaks based on changes in the environment of the pipeline. The use of an automated system based on developed UAVs could allow increased efficiency of tasks and reduced costs of entire operations.

The main advantages of helicopter-based remote sensing vehicles over electric multicopter vehicles include the much bigger payload capacity and flight time. In the category of helicopter-type mini-UAVs, WABIK has two main competitors, namely, Aeroscout Scout B1-100 [21] from Switzerland, which has an 18 kg payload capacity, and YAMAHA Rmax [22] from the USA, which has an 16 kg payload capacity. Flight endurance, payload options, operational range, or payload capacity are all parameters that are quite similar among the models, but WABIK is distinct from the others in terms of price, as it is much cheaper compared to the other models.

Work is currently underway to adapt WABIK to the agriculture industry. A spraying system equipped with two electric atomizers capable of atomizing 50 mm particles is under design. Two additional spray tanks could be mounted next to fuel tanks, and instead of the optoelectronic head, a diaphragm sprayer pump with a driver integrated with ground control station software could be installed at the front. This way, the spraying system could be controlled from the same ground control station by the same operator.

## Figures and Tables

**Table 1:** International classification of unmanned aerial vehicles (UAVs) [13].

Category	MTOW [kg]	Range [km]	Maximum Ceiling [m]
Micro	<5	<10	250
Mini	<25/30/150	<10	150/250/300
Short Range	25 ÷ 150	10 ÷ 30	3000
Medium Range	50 ÷ 250	30 ÷ 70	3000
Long Range	>250	>70	>3000

**Table 2:** Available control modes implemented in autopilot [16].

No.	Control Modes	Controller Number
1	CIC Attitude	1, 2, 3
2	CIC Position	1, 2, 3, 5, 6, optional 4
3	CIC Velocity	1, 2, 3, 5, 6, optional 4
4	CIC Altitude	1, 2, 3, 4
5	Full CIC	1, 2, 3, 4, 5, 6
